# Prostaglandin F2 receptor negative regulator as a potential target for chimeric antigen receptor-T cell therapy for glioblastoma

**DOI:** 10.1007/s00262-025-03979-4

**Published:** 2025-03-06

**Authors:** Hideki Kuroda, Noriyuki Kijima, Tetsuro Tachi, Shunya Ikeda, Koki Murakami, Tomoyoshi Nakagawa, Moto Yaga, Kanji Nakagawa, Reina Utsugi, Ryuichi Hirayama, Yoshiko Okita, Naoki Kagawa, Naoki Hosen, Haruhiko Kishima

**Affiliations:** 1 Department of Neurosurgery, Graduate School of Medicine, Osaka University, 2-2 Yamadaoka, Suita, 5650871 Japan; 2 World Premier International Immunology Frontier Research Center, Osaka University, Suita, Osaka Japan; 3 Department of Respiratory Medicine, Osaka General Hospital, Osaka, Osaka Japan; 4 Department of Hematology and Oncology, Graduate School of Medicine, Osaka University, 2-2 Yamadaoka, Suita, 5650871 Japan

**Keywords:** CAR-T cell therapy, Glioblastoma (GBM), PTGFRN, Expression cloning, Monoclonal antibodies

## Abstract

**Background:**

Chimeric antigen receptor (CAR)-T cell therapy targeting novel glioblastoma (GBM)-specific cell surface antigens is a promising approach. However, transcriptome analyses have revealed few GBM-specific target antigens.

**Methods:**

A library of monoclonal antibodies (mAbs) against tumor cell lines derived from patients with GBM was generated. mAbs reacting with tumor cells in resected tissues from patients with GBM but not with nonmalignant human brain cells were detected. The antigens that were recognized were identified through expression cloning. CAR-T cells derived from a candidate mAb were generated, and their functionality was tested in vitro and in vivo.

**Results:**

Approximately 3,200 clones were established. Among them, 5E17 reacted with tumor cells in six of seven patients with GBM, but not with nonmalignant human brain cells. Prostaglandin F2 receptor negative regulator (PTGFRN) was identified as an antigen recognized by 5E17. CAR-T cells derived from 5E17 produced cytokines and exerted cytotoxicity upon co-culture with tumor cells from patients with GBM. Furthermore, intracranial injection of 5E17-CAR-T cells demonstrated antitumor effects in an orthotopic xenograft murine model with patient-derived GBM cells.

**Conclusions:**

Cell surface PTGFRN is a candidate target for intracranial CAR-T cell therapy for GBM. On-target off-tumor toxicity in alternative normal tissues needs to be carefully tested.

**Supplementary Information:**

The online version contains supplementary material available at 10.1007/s00262-025-03979-4.

## Introduction

Glioblastoma (GBM) is the most common malignant brain tumor in adults. Despite intensive treatment with surgery, chemotherapy, and radiation, GBM remains incurable, with a median survival period of approximately 15–20 months [1, 2]. Therefore, more effective treatments are required, and various therapies for GBM are being tested or are under development [3–5].

Chimeric antigen receptor (CAR)-T–cell therapy is effective for B-cell malignancies, and recent studies have suggested its benefits for central nervous system malignancies, including GBM [6, 7]. In particular, CAR-T cells directed to disialoganglioside 2 (GD2) have generated promising results against H3-K27M+diffuse midline gliomas and neuroblastomas [8, 9]. In addition, epidermal growth factor (EGF) receptor variant III (EGFRvIII), human EGF receptor 2 (HER2), interleukin-13 receptor α2 chain (IL13Rα2), and B7-H3 have been used as target antigens for CAR-T cell therapy for GBM [7, 10–14]. However, to date, no CAR-T cells have been approved for GBM, partly because these antigens are not expressed in all heterogeneous GBM cells [4, 11, 15]. Furthermore, immunotherapy with a single target antigen often causes immune evasion through antigen loss in tumor cells [10, 16]. Therefore, more target antigens for GBM are required, although it is difficult to identify new cell surface molecules specifically expressed in GBM cells from transcriptome data.

To identify the cell surface antigens specifically expressed on tumor cells, we used a monoclonal antibody-based strategy and identified two multiple myeloma-specific antigens that could not be identified using transcriptome analysis [17, 18]. Previously, we applied the same strategy to GBM and identified B7-H3 as a GBM-specific antigen, indicating that screening is effective for GBM [19]. This study aimed to continue the screening using additional GBM samples and identify prostaglandin F2 receptor negative regulator (PTGFRN) as a potential target for CAR-T cell therapy for GBM.

## Materials and methods

### Clinical samples and tumor cells

Tumor tissues from seven patients with GBM (NP3022, NP7722, NP2523, NP3322, NP5223, NP423, and NP2023) and nonmalignant brain tissues from six patients with refractory epilepsy who underwent surgical resection (NP8222, NP7822, NP7022, NP2423, NP4923, and NP3523) were used after obtaining informed consent (Table 1). This study adhered to the tenets of the Declaration of Helsinki (2013, as amended) and was approved by the Ethics Review Committee of Osaka University Graduate School of Medicine (Suita, Osaka, Japan) (approval number: 20561).

**Table 1:** Clinical characteristics of patients and patient-derived tumor cell lines

	Age	Sex	IDH	pTERT	MGMT	Diagnosis
GDC519	67	M	Wild type	Mutant	Unmethylated	GBM
GDC3320	48	M	Wild type	Mutant	Unmethylated	GBM
GDC521	55	M	Wild type	Mutant	Unmethylated	GBM
GDC1121	72	F	Wild type	Mutant	Unmethylated	GBM
GDC3621	49	M	Wild type	Mutant	Unmethylated	GBM
GDC4221	45	M	Wild type	Mutant	Methylated	GBM
GDC4521	81	F	Wild type	Mutant	Unmethylated	GBM
GDC322	71	M	Wild type	Mutant	Unmethylated	GBM
GDC422	65	M	Wild type	Mutant	Unmethylated	GBM
GDC622	86	M	Wild type	Mutant	Methylated	GBM
GDC 1122	74	M	Wild type	Mutant	Methylated	GBM
GDC 1822	78	M	Wild type	Mutant	Unmethylated	GBM
GDC2822	68	F	Wild type	Mutant	Unmethylated	GBM
GDC2922	88	F	Wild type	Wild type	Unmethylated	GBM
GDC3222	72	F	Wild type	Mutant	Unmethylated	GBM
GDC 123	50	M	Wild type	Mutant	Methylated	GBM
GDC 1523	61	M	Wild type	Mutant	Unmethylated	GBM
GDC 1723	58	F	Wild type	Mutant	Unmethylated	GBM
GDC2223	44	M	Wild type	Mutant	Unmethylated	GBM
NP3022	56	M	Wild type	Mutant	Unmethylated	GBM
NP3322	79	F	Wild type	Wild type	Methylated	GBM
NP7722	84	F	Wild type	Wild type	Unmethylated	GBM
NP423	50	M	Wild type	Mutant	Methylated	GBM
NP2023	61	M	Wild type	Mutant	Unmethylated	GBM
NP5323	58	F	Wild type	Mutant	Unmethylated	GBM
NP2523	58	M	Wild type	Mutant	Methylated	GBM
NP3522	20	M	N/A	N/A	N/A	Epilepsy
NP7022	27	F	N/A	N/A	N/A	Epilepsy
NP7822	27	M	N/A	N/A	N/A	Epilepsy
NP8222	42	M	N/A	N/A	N/A	Epilepsy
NP2423	26	F	N/A	N/A	N/A	Epilepsy
NP4923	47	F	N/A	N/A	N/A	Epilepsy

Patient-derived tumor cell lines (PDTCs) were generated and cultured in a serum-free culture medium containing EGF and basic fibroblast growth factor, as previously reported [19]. In this study, PDTCs established from 19 patients (GDC519, GDC3320, GDC521, GDC1121, GDC3621, GDC4221, GDC4521, GDC322, GDC422, GDC622, GDC1122, GDC1822, GDC2822, GDC2922, GDC3222, GDC123, GDC1523, GDC1723, and GDC2223) were used (Table 1). U87MG, T98G and U251 cell lines were purchased from the American Type Culture Collection (Manassas, VA, USA) and were confirmed to have no mycoplasma contamination. The SP2/0 mouse myeloma cell line was kindly gifted by I. Weissman (Stanford University).

### Animal experiments

All animal experiments were approved by the Institutional Animal Care and Use Committee at Osaka University Graduate School of Medicine (approval numbers: 03-071-000 and 04-028-002) and were performed according to the animal use guidelines of the Animal Experiment Committee of Osaka University Graduate School of Medicine. No statistical method was used to determine the sample size.

### Generation of anti-glioblastoma (GBM) monoclonal antibodies (mAbs)

We immunized 6-week-old BALB/c mice (CLEA Japan, Tokyo, Japan) with 1 × 10^6^ GBM PDTCs every 1–2 weeks four times by injection into their right footpads. The PDTCs used for immunization were GDC519, GDC3320, GDC521, GDC1121, GDC3621 and GDC622. Lymphocytes from the right popliteal lymph nodes were fused to SP2/0 mouse myeloma cells using polyethylene glycol solution (Roche Applied Science, Penzberg, Germany). Hybridoma cells fused to lymphocytes and SP2/0 cells were suspended in 96-well plates and incubated for 2–4 weeks. To select hybridomas producing monoclonal antibodies (mAbs) that react with GBM, GBM PDTCs which used for immunization were first reacted with Human Serum AB (Gemini Bio-Products, West Sacramento, CA, USA) to block nonspecific binding. Subsequently, the GBM PDTCs were incubated with hybridoma supernatants and then with a PE-conjugated anti-mouse IgG antibody (eBioscience, San Diego, CA, USA) and analyzed by flow cytometry. Hybridoma clones that produced mAbs that reacted with GBM cells were frozen and stored for further analysis. Data were analyzed using the FlowJo software version 10.10.0 (Tree Star, Inc., Ashland, OR, USA).

### Flow cytometry

Tissue samples from patients with GBM and epilepsy were dissociated using the Brain Tumor Dissociation Kit (Miltenyi Biotec, Bergisch Gladbach, Germany) and GentleMACS Octo Dissociator with Heaters (Miltenyi Biotec), following the manufacturer’s protocol. The resulting single-cell suspensions were filtered at 40 μm with Hank’s Balanced Salt Solution containing Ca^2+^ and Mg^2+^ (Sigma-Aldrich Corp., St. Louis, MO, USA). Subsequently, we collected the cells by centrifugation for 5 min at 300 × *g*, lysed the red blood cells with ACK buffer (Thermo Fisher Scientific, Inc.) for 5 min on ice, and removed the supernatant. We resuspended the cells in a staining medium (2% fetal bovine serum + phosphate-buffered saline [PBS]) at 2 × 10^6^ /mL. The cells were incubated with Human Serum AB (Gemini Bio-Products) for 5 min to prevent nonspecific binding. Subsequently, the cells were incubated with the hybridoma supernatant for 30 min and with PE-conjugated anti-mouse IgG antibody. After washing with PBS, the cells were stained with anti-CD31-APC (eBioscience), anti-CD45-PEcy7 (BioLegend, San Diego, CA, USA), CD90-FITC (BioLegend), and propidium iodide and analyzed by flow cytometry using a FACS Canto II (Becton, Dickinson and Company). GBM tumor cells were identified as CD31-CD45-CD90 + cells because CD90 is strongly positive in most GBM cells [20]. In patients with epilepsy, we examined CD31– and CD45– cells, most of which were neurons, astrocytes, and oligodendrocytes [20, 21]. Data were analyzed using the FlowJo software (Tree Star).

### Expression cloning

Expression cloning was performed, as previously reported [19, 22]. A cDNA library was generated from GBM PDTCs (GDC40 used as previously reported) using Superscript Choice System (Invitrogen) and linked with a BstXI adaptor. Complementary DNA fragments (1.0–5.0 kb) were selected using CHROMA SPIN columns (Takara Bio Inc.), purified by agarose gel electrophoresis, and then subcloned into retroviral vector pMXs. The cDNA library constructed from the GBM PDTCs cells was transduced into Ba/F3 cells, a mouse pro-B cell line derived from the C3H strain, which were purchased from RIKEN BioResource Center (Ibaraki, Japan). We sorted and expanded the Ba/F3 cells that reacted with 5E17 four times by fluorescence-activated cell sorting (FACS). Then, DNA from the enriched Ba/F3 was extracted using DNeasy Blood and Tissue kits (Qiagen, GmbH), amplified by PCR, and analyzed by Sanger sequencing.

### Generation of prostaglandin F2 receptor negative regulator (PTGFRN)-overexpressing or PTGFRN-deficient cell lines

Ba/F3 cells were retrovirally transduced with PTGFRN cDNA to generate PTGFRN-overexpressing cells. The retrovirus was generated by transfecting 293 T cells with the retroviral vector, gag-pol, and VSV-G envelope plasmid using Lipofectamine 2000 (Thermo Fisher Scientific, Inc.) and harvested after 48 h. Additionally, we established PTGFRN knockout (KO) U87MG cells using the CRISPR Cas9 system. We generated crRNAs using the Integrated DNA Technologies (IDT) design tool. The target sequence was GACGTGCGCCTCGACACCGT. We generated an RNP complex consisting of a mixture of crRNA, tracrRNA (IDT, catalog no. 1072533), and TrueCut Cas9 protein V2 (Thermo Fisher Scientific, Inc.). The RNP complex was then electroporated into U87MG cells (5 × 10^6^) by using a NEPA 21 electroporator (Nepa Gene, Ichikawa, Japan) [23]. Overexpression and KO of PTGFRN were confirmed using known anti-PTGFRN antibody (R&D systems, inc.) and flow cytometry.

### Development of chimeric antigen receptor (CAR)-T cells for the candidate antigen and control

Variable regions of 5E17 were amplified through 5’-RACE PCR using Smarter RACE PCR kits (Takara Bio, Kusatsu, Japan) and sequenced. The cDNAs of the heavy and kappa light chains were fused with CD28 and CD3ζ by overlapping PCR [24]. The CD19 CAR was constructed according to the reported sequences of the anti-CD19 mAb [25, 26]. Subsequently, the resulting CAR construct was integrated into the pMXs retroviral vector. Peripheral blood mononuclear cells (PBMCs) taken from a healthy adult male were activated with anti-CD3 (OKT3; eBioscience) and anti-CD28 (CD28.2; eBioscience) and cultured in the presence of recombinant human interleukin-2 (IL-2) (Shionogi Pharma, Osaka, Japan) at a final concentration of 100 IU/mL. Two days later, the PBMCs were transduced with the CAR retrovirus in the viral supernatants using RetroNectin (Takara Bio, Inc.). The transduced cells were cultured with IL-2 (100 IU/mL) for 10 days and analyzed to determine the transduction efficiency of the CAR. Cells were incubated with Human Serum AB (Gemini Bio-Products) for 5 min to prevent nonspecific binding. Subsequently, the cells were stained with goat anti-mouse F(ab′)2-Alexa Fluor 647 (Jackson ImmunoResearch Laboratories, Inc., West Grove, PA, USA) for 30 min and analyzed using a FACS Canto II. We used CD19 transduced T cells as a nonspecific control.

### Cytokine release assays

The 5E17 CAR-T cells and CD19-transduced T cells (control T cells) were co-cultured with the indicated target cells for 16 h. Cytokine production was measured using Quantikine ELISA kits (IL-2 and interferon gamma [IFN-γ]; R&D Systems, Inc.). Co-culture was performed with quintuplicate wells.

### ^***51***^***Cr cytotoxicity assay***

The cytotoxic ability of CAR-T cells was evaluated using a ^51^Cr release assay, as previously described [19]. Briefly, 1 × 10^6^ tumor cells were marked at 37 °C with 200 μCi of [^51^Cr] sodium chromate (GE Healthcare, Chicago, IL, USA) for 2 h. Subsequently, the target (1 × 10^4^) and effector cells were cultured together. The effector/target ratios were set at 3, 9, and 27. Four hours later, the amount of ^51^Cr released was measured using a gamma counter. The spontaneous and maximum amounts of ^51^Cr released were determined using the same volume of target cells in the culture medium or 1%Triton X-100. Percentage-specific lysis was calculated as follows: ([specific ^51^Cr release – spontaneous ^51^Cr release]/[maximum ^51^Cr release – spontaneous ^51^Cr release]) × 100.

### In vivo xenograft mouse models

Orthotopic xenografts were established with patient-derived GBM cells utilizing NOD/Shi-scid IL2Rγ KO mice (NOG) (CIEA, Kawasaki, Japan). The mice were anesthetized with isoflurane, the skull burr hole was opened using a drill, and 2 × 10^5^ GDC519 cells labeled with GFP/luciferase were injected into the right cerebrum using a stereotactic injector (Muromachi Kikai, Osaka, Japan). The injection point in the cerebrum was 1 mm forward of the bregma, 2 mm to the right, and 2 mm deep. Six days after tumor injection, GBM tumor development in mice was confirmed using an In Vivo Imaging System (IVIS) (PerkinElmer, Inc., Waltham, MA, USA) and 150 μL luciferin (Promega, Madison, WI, USA). Mice were randomized into two groups according to the fluorescence intensities of the GBM tumors. There were no significant differences in the fluorescence intensities between GBM tumors from mice treated with 5E17 CAR-T cells and those treated with control T cells. Seven days after tumor injection, 2 × 10^6^ 5E17 CAR-T or control T (CD19) cells were injected at the same injection point as the tumor cell injections. We did not inject IL2 in mice. Tumor volume was analyzed weekly using the IVIS. The mice were euthanized when they exhibited neurological symptoms. Mice in which excessive tumor growth was observed before CAR-T cell injection (> 8 × 10e7 p/s/cm^2^/sr in IVIS luminescence) were excluded from the analysis.

#### Statistical analyses

Statistical analyses were performed using the JMP software (version 16.0; SAS Institute, Cary, NC, USA). The Mann–Whitney U-test was used for group comparisons. Mouse survival was analyzed using Kaplan–Meier curves, and differences were assessed using log-rank tests. A *p* value < *0.05* was considered significant.

## Results

### Identification of 5E17 as a GBM-specific mAb

**Fig. 1:**
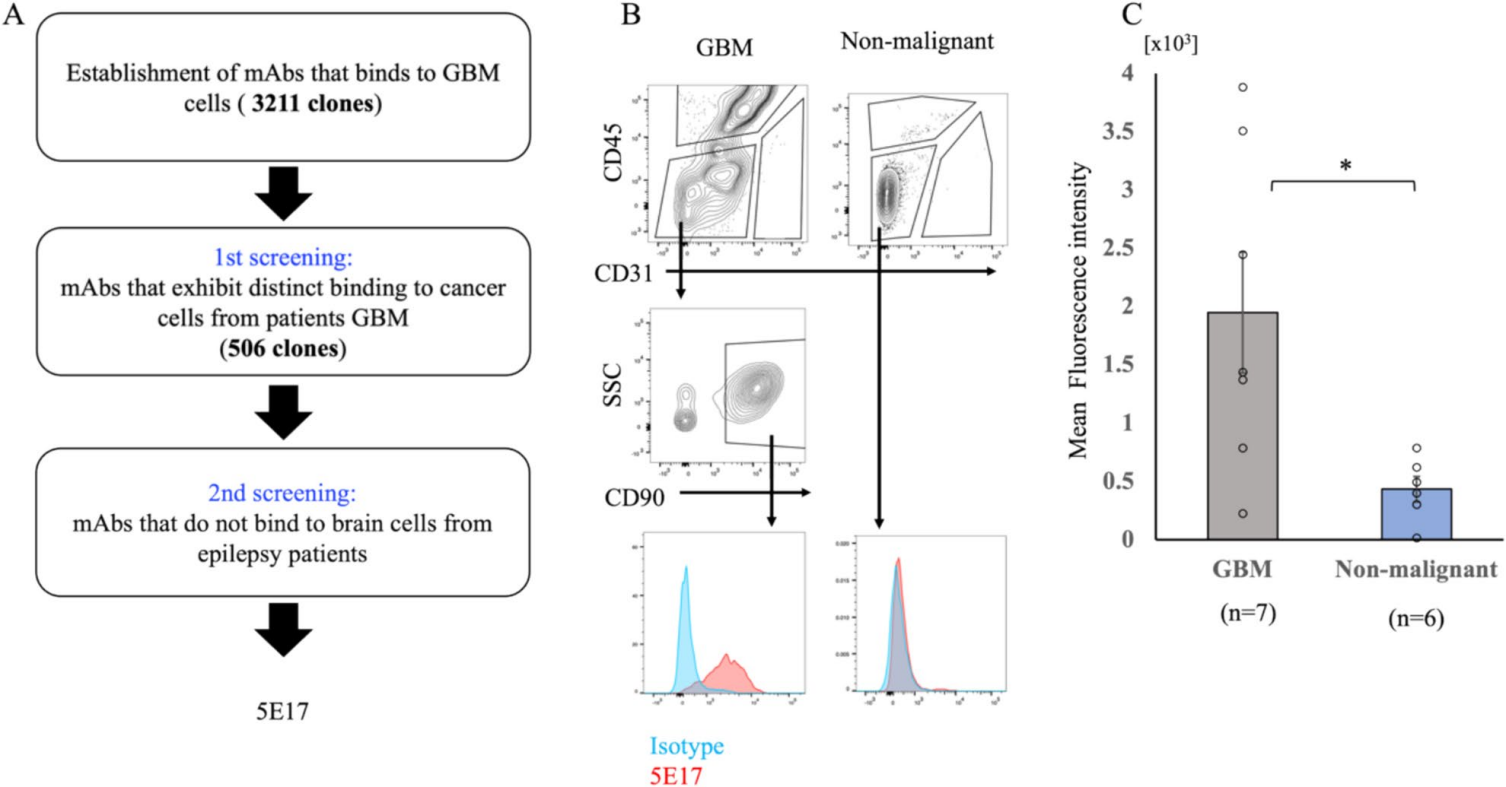
Identification of 5E17 as a GBM-specific mAb. **A** Strategy for the identification of the GBM-specific mAb 5E17. **B** Representative flow cytometry findings of 5E17 mAb bound to GBM and nonmalignant brain cells from patients. Analyses of live (PI-negative) cells are shown. The results of staining with the isotype or negative control instead of anti-5E17 mAb, CD31, CD45, or CD90 are shown to draw the gate for 5E17-positive cells. Analyses of the other patients are shown in Supplementary Fig. 2. Blue histogram indicates isotype control. SSC, side scatter. **C** Mean fluorescence intensity (means ± standard error of the mean) of flow cytometry findings bound to GBM (*n* = 7) and nonmalignant brain cells (*n* = 6) from patients. Each plot shows the mean fluorescence intensity. GBM cells were assessed as CD31-CD45-CD90 + cells, and nonmalignant tumor cells were assessed as CD31-CD45- cells. ^*^p < 0.05, calculated using a Mann–Whitney *U*-test

Mice were immunized with PDTCs, and 3211 mAbs that bound to GBM PDTCs were established. Next, the binding of these mAbs to CD45-CD31-CD90+GBM tumor cells in resected tissues from patients with GBM was examined using flow cytometry, and 506 mAbs that exhibited distinct binding to at least one resected tissue from patients with GBM [20] were selected. Finally, the binding of these candidate mAbs to CD45^−^CD31^−^ cells in nonmalignant cells obtained from patients with epilepsy was analyzed, and 3N5, 4F11, 5E17 were identified as a candidate GBM-specific mAbs. Among these, we chose 5E17 as the most promising mAb because 5E17 reacted with many GBM samples (Fig. 1A, Supplementary Fig. 1). Distinct binding of 5E17 was detected in three GBM cell lines (U87MG, T98G, and U251) and 18 of the 19 PDTCs (Supplementary Fig. 1). In two of seven primary GBM samples, 5E17 distinctly bounded to all CD45^−^CD31^−^CD90^+^ GBM tumor cells, whereas 5E17 also bounded to a subset of tumor cells in the other four GBM samples (Fig. 1B, C, Supplementary Fig. 2) [20]. In contrast, 5E17 did not react with CD45^−^CD31^−^ cells in nonmalignant cells (Fig. 1B, C, Supplementary Fig. 2). 5E17 reactivity in PDTCs and primary GBM samples did not differ in patients with pTERT mutation, but was higher in patients with unmethylated MGMT (Table 1, Supplementary Fig. 3).

### 5E17 recognized PTGFRN

**Fig. 2:**
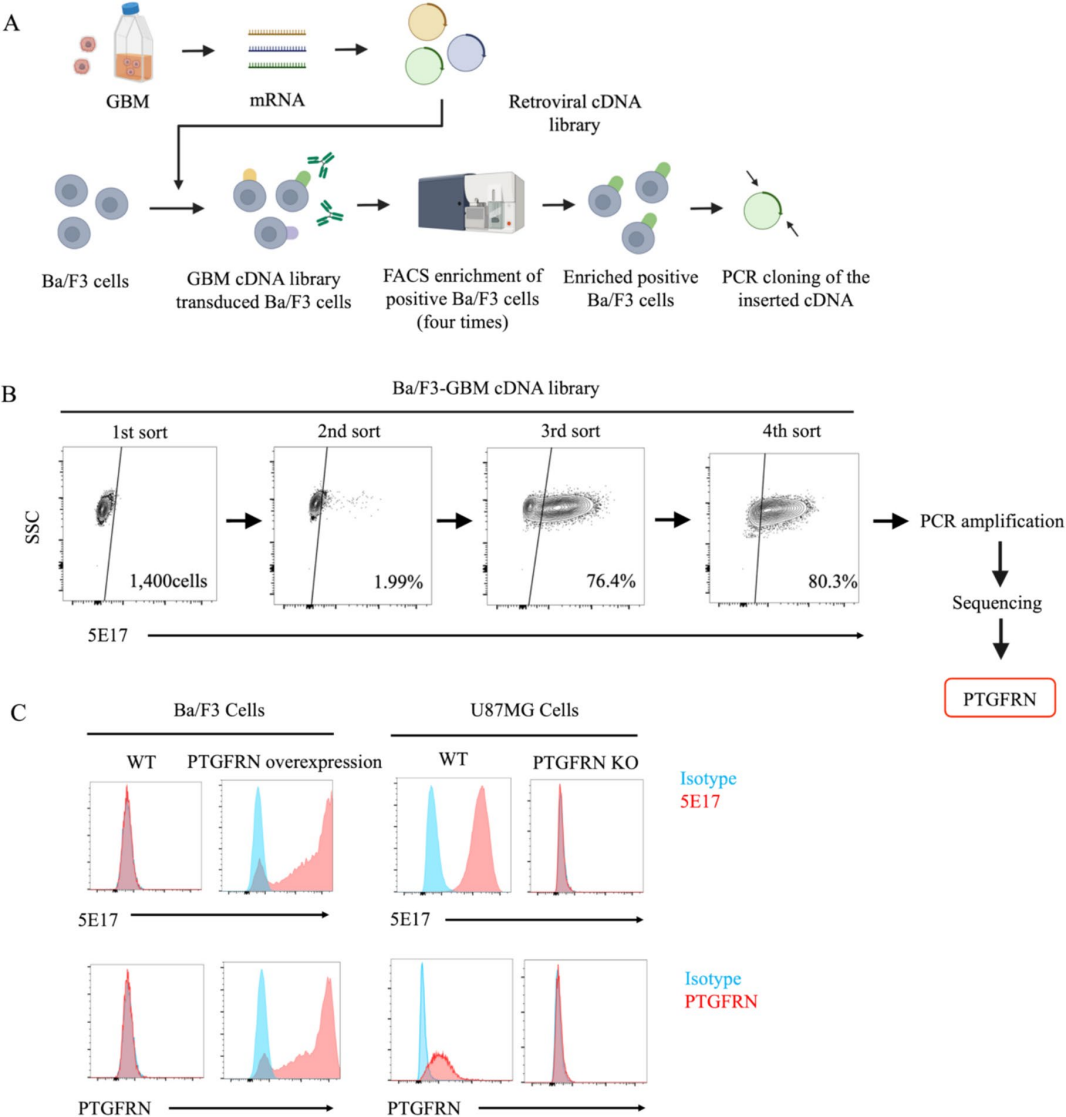
5E17 recognized PTGFRN. **A** Procedure for expression cloning to identify antigens recognized by 5E17 monoclonal antibody. **B** Flow cytometry plots showing the process of 5E17-positive cell enrichment in the expression cloning of the 5E17 antigen. SSC, side scatter. **C** Flow cytometry analysis of 5E17 (top row) and commercially available anti-PTGFRN antibody (R&D systems, inc.) (bottom row) reactivity to wild/5E17 overexpressed Ba/F3 cells and wild/PTGFRN KO U87MG cells. WT, wild type; KO, knock out

The antigen recognized by 5E17 was identified using expression cloning. A retroviral cDNA library, previously generated from GBM cells, was transduced into mouse Ba/F3 cells that did not react with 5E17 [19]. Transduced Ba/F3 cells were stained with 5E17, and cells expressing the antigen recognized by 5E17 were enriched using FACS. After four rounds of sorting, most Ba/F3 cells were positively stained with 5E17. Finally, the cDNA transduced into 5E17-reactive Ba/F3 cells was identified as PTGFRN, suggesting that 5E17 recognizes PTGFRN (Fig. 2A, B). The results confirmed that 5E17 cells consistently reacted with Ba/F3 cells transduced with human PTGFRN cDNA but not with PTGFRN KO U87MG cells (Fig. 2C).

### *T cells transduced with 5E17-derived CAR showed significant antitumor effect *in vitro

**Fig. 3:**
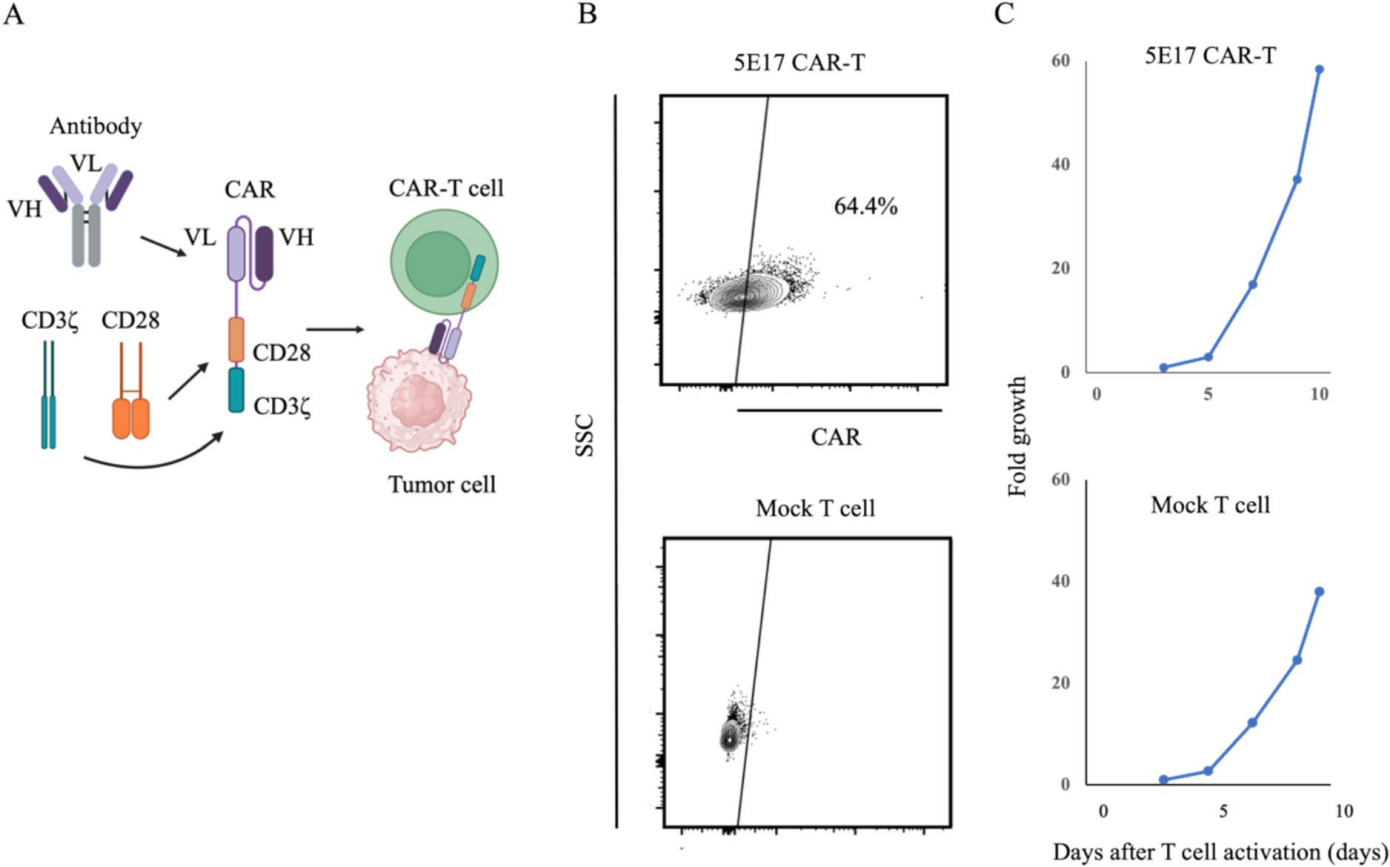
Development of CAR-T cells for the 5E17 antigen. **A** Establishment of CAR-T cells using the 5E17 monoclonal antibody in the indicated construct. VH, variable heavy; VL, variable light. **B** Flow cytometry plots of 5E17 CAR or Mock transduction efficiency in human T cells 10 days after CAR transduction. SSC, side scatter. **C** Growth of 5E17 CAR-T or Mock T cells during in vitro culture

**Fig. 4:**
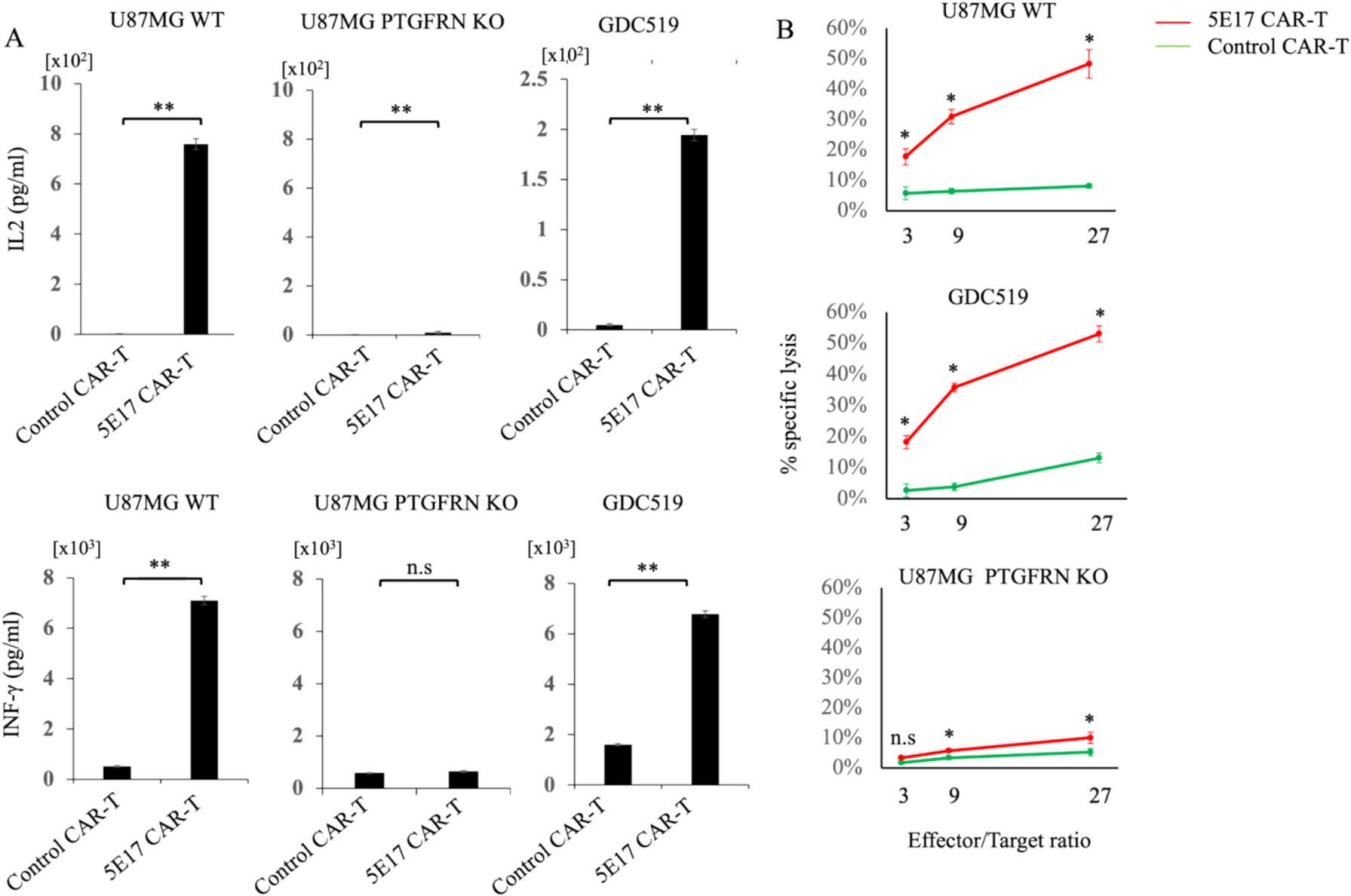
5E17 CAR-T cells are activated and have antitumor effects in vitro. **A** ELISA assay of IFN-γ and IL-2 released by 5E17 CAR-T cells or control CAR-T cells after co-culture with the indicated cells. Irrelevant antibody-transduced (CD19) T cells were used as controls. All experiments were performed in technical-quintuplicate wells. IFN-γ, interferon gamma; IL, interleukin; WT, wild type; KO, knock out. **B** ^51^Cr release assay to measure specific lysis of the indicated target cells by 5E17 or control CAR-T cells (CD19). All experiments were performed in technical-triplicate wells. Data are expressed as means ± standard error of the means. WT, wild type; KO, knock out. ^**^p < 0.01, ^*^p < 0.05, n.s, not significant, calculated using a Mann–Whitney *U*-test

An anti-5E17 CAR was designed consisting of 5E17 scFv and cytoplasmic regions of CD28 and CD3ζ. Thereafter, 5E17 CAR-T cells were established by transducing the CAR construct into T cells activated with CD3 and CD28 mAbs (Fig. 3A, B). The 5E17 CAR-T cells exponentially expanded (Fig. 3C). 5E17 CAR-T cells produced IFN-γ and IL-2 upon co-cultured with U87MG cells but not with PTGFRN KO U87MG cells. In addition, 5E17 CAR-T cells produced IFN-γ and IL-2 upon co-cultured with GDC519 in ELISA (Fig. 4A). Furthermore, 5E17 CAR-T cells exerted significant cytotoxicity against U87MG and GDC519 cells, but not against PTGFRN KO U87MG cells by ^51^Cr release assay (Fig. 4B). 5E17 CAR-T cells showed less cytotoxicity against GDC622, which expressed lower amounts of PTGFRN than GDC519 cells (Supplementary Fig. 4).

### Intracranial injection of 5E17 CAR-T cells eliminated GBM cells in orthotopic patient-derived xenograft model

**Fig. 5:**
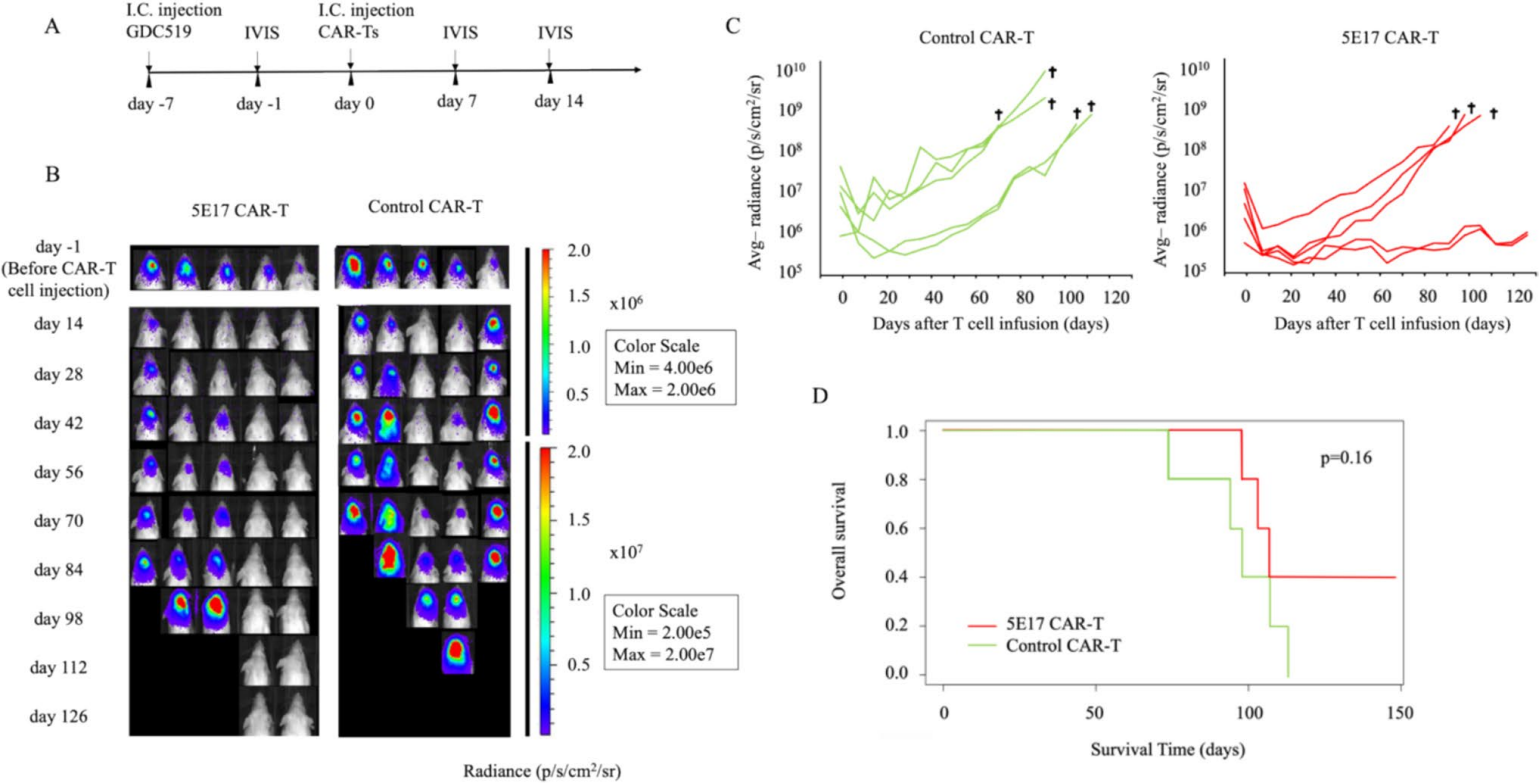
5E17 CAR-T cells are activated and have antitumor effects in vivo. **A** Experimental design. **B** Bioluminescence imaging of mice every 2 weeks after injection with 5E17 and control CAR-T cells (CD19) (*n* = 5 per group). p, photons; s, second; sr, steradian; Min, minimum; Max, maximum. **C** Quantification of the brain luminescence. Avg, average. **D** Kaplan–Meier curve of the mice infused with either 5E17 or control CAR-T cells (CD19). *p* = 0.16; calculated using a log rank test

An orthotopic xenograft model with patient-derived GBM cells (GDC519) was established by injecting luciferase-expressing GBM PDTCs into the right cerebrum of NOG mice [19]. Six days after injection of the GBM PDTCs, tumor development was confirmed by IVIS in all mice. The next day, 5E17 CAR-T cells or control T cells were injected into the tumor region (Fig. 5A). All mice treated with control T cells, including mice in which the initial tumor burden was low (rightmost of the control group in Fig. 5B), died by day 126 due to tumor progression (Fig. 5B–D). In contrast, two of the five mice treated with 5E17 CAR-T cells survived until day 126 and GBM tumors became undetectable (Fig. 5B, C). These results suggest that 5E17 CAR-T cells have antitumor activity, although the difference in overall survival between the mice treated with 5E17 CAR-T cells and those treated with control T cells was not significant (Fig. 5D).

## Discussion

PTGFRN belongs to the immunoglobulin superfamily and is a cell surface transmembrane protein. PTGFRN, also known as CD315, FPRP, CD9 partner-1, and EWI-F, is expressed in several types of cancer [27–29]. It interacts with tetraspanins, integrins, or ezrin-radixin-moesin proteins to regulate migration, angiogenesis, and cell polarity [30–33] and decreases the radiosensitivity of GBM cells [34]. Recently, silencing of PTGFRN in glioma cells in vitro downregulates cell proliferation, migration, invasion, cell cycle progression, and apoptosis, suggesting its oncogenic role in gliomas [27]. In addition, in vivo, PTGFRN has been shown to be involved in the tumor growth of lung cancer. Furthermore, an antibody–drug conjugate of PTGFRN conjugated with saporin has been shown to be effective against PTGFRN-positive cancer cells, such as epidermoid carcinoma, spindle carcinoma, and medulloblastoma [35, 36]. Although the functional significance of PTGFRN in GBM cells has not been tested in vivo.

In this study, PTGFRN was identified using monoclonal antibody-based screening. We chose mAb-based screening since it allows us to identify tumor-specific antigens including those generated by post-translational events such as conformational changes or modifications [17, 18]. However, it remains unclear whether 5E17 reacts with such a conformational epitope in the PTGFRN protein. Analysis of a public gene expression database [37] showed that PTGFRN expression was elevated in GBM tumors compared with normal brain tissues [34]. Since useful GBM-specific targets may have been missed by the mAb-based strategy, comprehensive transcriptome analysis, including single-cell RNAseq, of GBM and normal brain samples should be performed to identify more targets in future.

PTGFRN expression was scarcely detected in non-tumorous brain cells. Therefore, PTGFRN CAR-T cells may be safely administered intracranially or in the postsurgical resection cavity. Intracranial injection of CAR-T cells has recently been shown to be effective and safe in several clinical trials [10, 14, 38]. In preclinical animal studies, CAR-T cells were detected in systemic circulation even after intracranial injection, whereas circulating CAR-T cell numbers were lower than those after intravenous injection [39, 40]. Thus, it is important to carefully examine PTGFRN expression in vital organs. In future, testing the safety of PTGFRN-targeting therapy using mouse CAR-T cells targeting mouse PTGFRN will also be important.

The delivery route is another important issue related to the application of CAR-T cell therapy for brain tumors, with various options being considered, including intravenous, intraventricular, intrathecal, and intracavitary administration. A previous study has shown that CAR-T cells injected into the cavity displayed potential trafficking, even to distant sites within the brain [41]. In addition, recent clinical trials indicate that a combination of intracavitary with intraventricular or intravenous administration could be a suitable delivery route option with regard to the effectiveness and distribution of CAR-T cell therapy [42, 43].

In this study, we could not find a significant effect of PTGFRN CAR-T cells on OS, although we could observe cytotoxicity of PTGFRN CAR-T cells in vitro. We found that higher GBM expression of PTGFRN resulted in more cytotoxicity in vitro. Therefore, treating cases with higher PTGFRN expression and/or optimizing the delivery route of CAR-T cells such as via a combination of intracavitary and intraventricular/intravenous administration could lead to improvements in overall survival [42, 43]. However, further research is required as these factors were not assessed in the present study.

Finally, since 5E17 is a mouse antibody, it cannot be directly used in human patients. When used without any modification, the 5E17 scFv may cause an immune reaction. Therefore, it will be necessary to humanize the sequence of 5E17 scFv before proceeding to clinical trials. In conclusion, PTGFRN was identified as a potential target for CAR-T cell therapy for GBM and this study demonstrated its antitumor effects in vitro and in vivo. PTGFRN CAR-T cell therapy may be a potential novel therapeutic target for GBM.

## Supplementary Information

Below is the link to the electronic supplementary material.

Supplementary file1 (PDF 2012 KB)

Supplementary file2 (PDF 2352 KB)

Supplementary file3 (PDF 378 KB)

Supplementary file4 (PDF 1417 KB)

## Data Availability

The datasets generated during and/or analyzed during the current study are available from the corresponding author on reasonable request.

## Abbreviations

GBM: Glioblastoma
CAR: Chimeric antigen receptor
GD2: Disialoganglioside 2
EGF: Epidermal growth factor;
EGFRvII: EGF receptor variant III
HER2: Human EGF receptor 2
IL13Rα2: Interleukin-13 receptor α2 chain
PTGFRN: Prostaglandin F2 receptor negative regulator
PDTCs: Patient-derived tumor cell lines
mAbsa: Monoclonal antibodies
FACS: Fluorescence-activated cell sorting
PCR: Polymerase chain reaction
PTGFRN: Prostaglandin F2 receptor negative regulator
KO: Knockout
IDT: Integrated DNA Technologies
PBMCs: Peripheral blood mononuclear cells

## References

[1] Stupp R, Mason WP, van den Bent MJ, Weller M, Fisher B, Taphoorn MJ, Belanger K, Brandes AA, Marosi C, Bogdahn U, Curschmann J, Janzer RC, Ludwin SK, Gorlia T, Allgeier A, Lacombe D, Cairncross JG, Eisenhauer E, Mirimanoff RO (2005) Radiotherapy plus concomitant and adjuvant temozolomide for glioblastoma. N Engl J Med 352:987–996. 10.1056/NEJMoa043330 15758009 10.1056/NEJMoa043330

[2] Stupp R, Taillibert S, Kanner AA, Kesari S, Steinberg DM, Toms SA, Taylor LP, Lieberman F, Silvani A, Fink KL, Barnett GH, Zhu JJ, Henson JW, Engelhard HH, Chen TC, Tran DD, Sroubek J, Tran ND, Hottinger AF, Landolfi J, Desai R, Caroli M, Kew Y, Honnorat J, Idbaih A, Kirson ED, Weinberg U, Palti Y, Hegi ME, Ram Z (2015) Maintenance therapy with tumor-treating fields plus temozolomide vs temozolomide alone for glioblastoma: a randomized clinical trial. JAMA 314:2535–2543. 10.1001/jama.2015.16669 26670971 10.1001/jama.2015.16669

[3] Montoya ML, Kasahara N, Okada H (2020) Introduction to immunotherapy for brain tumor patients: challenges and future perspectives. Neurooncol Pract 7:465–476. 10.1093/nop/npaa007 33014387 10.1093/nop/npaa007 PMC7516091

[4] Tang X, Zhao S, Zhang Y, Wang Y, Zhang Z, Yang M, Zhu Y, Zhang G, Guo G, Tong A, Zhou L (2019) B7–H3 as a novel CAR-T therapeutic target for glioblastoma. Mol Ther Oncolytics 14:279–287. 10.1016/j.omto.2019.07.002 31485480 10.1016/j.omto.2019.07.002 PMC6713854

[5] Barros LRC, Couto SCF, da Silva SD, Paixão EA, Cardoso F, da Silva VJ, Klinger P, Ribeiro P, Rós FA, Oliveira TGM, Rego EM, Ramos RN, Rocha V (2022) Systematic review of available CAR-T cell trials around the world. Cancers. 10.3390/cancers14112667 35681646 10.3390/cancers14112667 PMC9179563

[6] June CH, Sadelain M (2018) Chimeric antigen receptor therapy. N Engl J Med 379:64–73. 10.1056/NEJMra1706169 29972754 10.1056/NEJMra1706169 PMC7433347

[7] O’Rourke DM, Nasrallah MP, Desai A, Melenhorst JJ, Mansfield K, Morrissette JJD, Martinez-Lage M, Brem S, Maloney E, Shen A, Isaacs R, Mohan S, Plesa G, Lacey SF, Navenot JM, Zheng Z, Levine BL, Okada H, June CH, Brogdon JL, Maus MV (2017) A single dose of peripherally infused EGFRvIII-directed CAR T cells mediates antigen loss and induces adaptive resistance in patients with recurrent glioblastoma. Sci Transl Med. 10.1126/scitranslmed.aaa0984 28724573 10.1126/scitranslmed.aaa0984 PMC5762203

[8] Majzner RG, Ramakrishna S, Yeom KW, Patel S, Chinnasamy H, Schultz LM, Richards RM, Jiang L, Barsan V, Mancusi R (2022) GD2-CAR T cell therapy for H3K27M-mutated diffuse midline gliomas. Nature 603:934–941 35130560 10.1038/s41586-022-04489-4 PMC8967714

[9] Louis CU, Savoldo B, Dotti G, Pule M, Yvon E, Myers GD, Rossig C, Russell HV, Diouf O, Liu E, Liu H, Wu MF, Gee AP, Mei Z, Rooney CM, Heslop HE, Brenner MK (2011) Antitumor activity and long-term fate of chimeric antigen receptor-positive T cells in patients with neuroblastoma. Blood 118:6050–6056. 10.1182/blood-2011-05-354449 21984804 10.1182/blood-2011-05-354449 PMC3234664

[10] Brown CE, Badie B, Barish ME, Weng L, Ostberg JR, Chang WC, Naranjo A, Starr R, Wagner J, Wright C, Zhai Y, Bading JR, Ressler JA, Portnow J, D’Apuzzo M, Forman SJ, Jensen MC (2015) Bioactivity and safety of IL13Rα2-redirected chimeric antigen receptor CD8+ T cells in patients with recurrent glioblastoma. Clin Cancer Res 21:4062–4072. 10.1158/1078-0432.Ccr-15-0428 26059190 10.1158/1078-0432.CCR-15-0428 PMC4632968

[11] Lin YJ, Mashouf LA, Lim M (2022) CAR T cell therapy in primary brain tumors: current investigations and the future. Front Immunol 13:817296. 10.3389/fimmu.2022.817296 35265074 10.3389/fimmu.2022.817296 PMC8899093

[12] Goff SL, Morgan RA, Yang JC, Sherry RM, Robbins PF, Restifo NP, Feldman SA, Lu YC, Lu L, Zheng Z, Xi L, Epstein M, McIntyre LS, Malekzadeh P, Raffeld M, Fine HA, Rosenberg SA (2019) Pilot trial of adoptive transfer of chimeric antigen receptor-transduced T cells targeting EGFRvIII in patients with glioblastoma. J Immunother 42:126–135. 10.1097/cji.0000000000000260 30882547 10.1097/CJI.0000000000000260 PMC6691897

[13] Shen L, Li H, Bin S, Li P, Chen J, Gu H, Yuan W (2019) The efficacy of third generation anti-HER2 chimeric antigen receptor T cells in combination with PD1 blockade against malignant glioblastoma cells. Oncol Rep 42:1549–1557. 10.3892/or.2019.7263 31524276 10.3892/or.2019.7263

[14] Tang X, Wang Y, Huang J, Zhang Z, Liu F, Xu J, Guo G, Wang W, Tong A, Zhou L (2021) Administration of B7–H3 targeted chimeric antigen receptor-T cells induce regression of glioblastoma. Signal Transduct Target Ther 6:125. 10.1038/s41392-021-00505-7 33767145 10.1038/s41392-021-00505-7 PMC7994554

[15] Eskilsson E, Rosland GV, Talasila KM, Knappskog S, Keunen O, Sottoriva A, Foerster S, Solecki G, Taxt T, Jirik R, Fritah S, Harter PN, Välk K, Al Hossain J, Joseph JV, Jahedi R, Saed HS, Piccirillo SG, Spiteri I, Leiss L, Euskirchen P, Graziani G, Daubon T, Lund-Johansen M, Enger P, Winkler F, Ritter CA, Niclou SP, Watts C, Bjerkvig R, Miletic H (2016) EGFRvIII mutations can emerge as late and heterogenous events in glioblastoma development and promote angiogenesis through Src activation. Neuro Oncol 18:1644–1655. 10.1093/neuonc/now113 27286795 10.1093/neuonc/now113 PMC5791772

[16] Chuntova P, Downey KM, Hegde B, Almeida ND, Okada H (2018) Genetically engineered T-cells for malignant glioma: overcoming the barriers to effective immunotherapy. Front Immunol 9:3062. 10.3389/fimmu.2018.03062 30740109 10.3389/fimmu.2018.03062 PMC6357938

[17] Hosen N, Matsunaga Y, Hasegawa K, Matsuno H, Nakamura Y, Makita M, Watanabe K, Yoshida M, Satoh K, Morimoto S, Fujiki F, Nakajima H, Nakata J, Nishida S, Tsuboi A, Oka Y, Manabe M, Ichihara H, Aoyama Y, Mugitani A, Nakao T, Hino M, Uchibori R, Ozawa K, Baba Y, Terakura S, Wada N, Morii E, Nishimura J, Takeda K, Oji Y, Sugiyama H, Takagi J, Kumanogoh A (2017) The activated conformation of integrin β(7) is a novel multiple myeloma-specific target for CAR T cell therapy. Nat Med 23:1436–1443. 10.1038/nm.4431 29106400 10.1038/nm.4431

[18] Hasegawa K, Ikeda S, Yaga M, Watanabe K, Urakawa R, Iehara A, Iwai M, Hashiguchi S, Morimoto S, Fujiki F, Nakajima H, Nakata J, Nishida S, Tsuboi A, Oka Y, Yoshihara S, Manabe M, Ichihara H, Mugitani A, Aoyama Y, Nakao T, Hirose A, Hino M, Ueda S, Takenaka K, Masuko T, Akashi K, Maruno T, Uchiyama S, Takamatsu S, Wada N, Morii E, Nagamori S, Motooka D, Kanai Y, Oji Y, Nakagawa T, Kijima N, Kishima H, Ikeda A, Ogino T, Shintani Y, Kubo T, Mihara E, Yusa K, Sugiyama H, Takagi J, Miyoshi E, Kumanogoh A, Hosen N (2022) Selective targeting of multiple myeloma cells with a monoclonal antibody recognizing the ubiquitous protein CD98 heavy chain. Sci Transl Med 14:eaax7706. 10.1126/scitranslmed.aax7706 35171652 10.1126/scitranslmed.aax7706

[19] Nakagawa T, Kijima N, Hasegawa K, Ikeda S, Yaga M, Wibowo T, Tachi T, Kuroda H, Hirayama R, Okita Y, Kinoshita M, Kagawa N, Kanemura Y, Hosen N, Kishima H (2023) Identification of glioblastoma-specific antigens expressed in patient-derived tumor cells as candidate targets for chimeric antigen receptor T cell therapy. Neurooncol Adv 5:vdac177. 10.1093/noajnl/vdac177 36601313 10.1093/noajnl/vdac177 PMC9798403

[20] Dirkse A, Golebiewska A, Buder T, Nazarov PV, Muller A, Poovathingal S, Brons NHC, Leite S, Sauvageot N, Sarkisjan D, Seyfrid M, Fritah S, Stieber D, Michelucci A, Hertel F, Herold-Mende C, Azuaje F, Skupin A, Bjerkvig R, Deutsch A, Voss-Böhme A, Niclou SP (2019) Stem cell-associated heterogeneity in Glioblastoma results from intrinsic tumor plasticity shaped by the microenvironment. Nat Commun 10:1–16. 10.1038/s41467-019-09853-z 30992437 10.1038/s41467-019-09853-z PMC6467886

[21] Darmanis S, Sloan SA, Zhang Y, Enge M, Caneda C, Shuer LM, Hayden Gephart MG, Barres BA, Quake SR (2015) A survey of human brain transcriptome diversity at the single cell level. Proc Natl Acad Sci U S A 112:7285–7290. 10.1073/pnas.1507125112 26060301 10.1073/pnas.1507125112 PMC4466750

[22] Kitamura T, Onishi M, Kinoshita S, Shibuya A, Miyajima A, Nolan GP (1995) Efficient screening of retroviral cDNA expression libraries. Proc Natl Acad Sci U S A 92:9146–9150. 10.1073/pnas.92.20.9146 7568090 10.1073/pnas.92.20.9146 PMC40941

[23] Yoshikawa T, Wu Z, Inoue S, Kasuya H, Matsushita H, Takahashi Y, Kuroda H, Hosoda W, Suzuki S, Kagoya Y (2022) Genetic ablation of PRDM1 in antitumor T cells enhances therapeutic efficacy of adoptive immunotherapy. Blood 139:2156–2172. 10.1182/blood.2021012714 34861037 10.1182/blood.2021012714

[24] Maher J, Brentjens RJ, Gunset G, Rivière I, Sadelain M (2002) Human T-lymphocyte cytotoxicity and proliferation directed by a single chimeric TCRzeta /CD28 receptor. Nat Biotechnol 20:70–75. 10.1038/nbt0102-70 11753365 10.1038/nbt0102-70

[25] Kowolik CM, Topp MS, Gonzalez S, Pfeiffer T, Olivares S, Gonzalez N, Smith DD, Forman SJ, Jensen MC, Cooper LJ (2006) CD28 costimulation provided through a CD19-specific chimeric antigen receptor enhances in vivo persistence and antitumor efficacy of adoptively transferred T cells. Cancer Res 66:10995–11004. 10.1158/0008-5472.Can-06-0160 17108138 10.1158/0008-5472.CAN-06-0160

[26] Terakura S, Yamamoto TN, Gardner RA, Turtle CJ, Jensen MC, Riddell SR (2012) Generation of CD19-chimeric antigen receptor modified CD8+ T cells derived from virus-specific central memory T cells. Blood 119:72–82. 10.1182/blood-2011-07-366419 22031866 10.1182/blood-2011-07-366419 PMC3251238

[27] Mala U, Baral TK, Somasundaram K (2022) Integrative analysis of cell adhesion molecules in glioblastoma identified prostaglandin F2 receptor inhibitor (PTGFRN) as an essential gene. BMC Cancer 22:642. 10.1186/s12885-022-09682-2 35690717 10.1186/s12885-022-09682-2 PMC9188228

[28] Liu J, Ji Y, Weng X, Shao W, Zhao J, Chen H, Shen L, Wang F, Meng Q, Wu X, Wang X, Ou Q, Ke H (2023) Immune microenvironment analysis and novel biomarkers of early-stage lung adenocarcinoma evolution. Front Oncol 13:1150098. 10.3389/fonc.2023.1150098 37427097 10.3389/fonc.2023.1150098 PMC10328385

[29] Kempska J, Oliveira-Ferrer L, Grottke A, Qi M, Alawi M, Meyer F, Borgmann K, Hamester F, Eylmann K, Rossberg M, Smit DJ, Jücker M, Laakmann E, Witzel I, Schmalfeldt B, Müller V, Legler K (2023) Impact of AKT1 on cell invasion and radiosensitivity in a triple negative breast cancer cell line developing brain metastasis. Front Oncol 13:1129682. 10.3389/fonc.2023.1129682 37483521 10.3389/fonc.2023.1129682 PMC10358765

[30] Chambrion C, Le Naour F (2010) The tetraspanins CD9 and CD81 regulate CD9P1-induced effects on cell migration. PLoS ONE 5:e11219. 10.1371/journal.pone.0011219 20574531 10.1371/journal.pone.0011219 PMC2888588

[31] Sala-Valdés M, Ursa A, Charrin S, Rubinstein E, Hemler ME, Sánchez-Madrid F, Yáñez-Mó M (2006) EWI-2 and EWI-F link the tetraspanin web to the actin cytoskeleton through their direct association with ezrin-radixin-moesin proteins. J Biol Chem 281:19665–19675. 10.1074/jbc.M602116200 16690612 10.1074/jbc.M602116200

[32] Colin S, Guilmain W, Creoff E, Schneider C, Steverlynck C, Bongaerts M, Legrand E, Vannier JP, Muraine M, Vasse M, Al-Mahmood S (2011) A truncated form of CD9-partner 1 (CD9P-1), GS-168AT2, potently inhibits in vivo tumour-induced angiogenesis and tumour growth. Br J Cancer 105:1002–1011. 10.1038/bjc.2011.303 21863033 10.1038/bjc.2011.303 PMC3185932

[33] Charrin S, Le Naour F, Oualid M, Billard M, Faure G, Hanash SM, Boucheix C, Rubinstein E (2001) The major CD9 and CD81 molecular partner. Identification and characterization of the complexes. J Biol Chem 276:14329–14337. 10.1074/jbc.M011297200 11278880 10.1074/jbc.M011297200

[34] Aguila B, Morris AB, Spina R, Bar E, Schraner J, Vinkler R, Sohn JW, Welford SM (2019) The Ig superfamily protein PTGFRN coordinates survival signaling in glioblastoma multiforme. Cancer Lett 462:33–42. 10.1016/j.canlet.2019.07.018 31377205 10.1016/j.canlet.2019.07.018 PMC6705426

[35] Guilmain W, Colin S, Legrand E, Vannier JP, Steverlynck C, Bongaerts M, Vasse M, Al-Mahmood S (2011) CD9P-1 expression correlates with the metastatic status of lung cancer, and a truncated form of CD9P-1, GS-168AT2, inhibits in vivo tumour growth. Br J Cancer 104:496–504. 10.1038/sj.bjc.6606033 21206492 10.1038/sj.bjc.6606033 PMC3049554

[36] Marquez J, Dong J, Dong C, Tian C, Serrero G (2021) Identification of Prostaglandin F2 Receptor Negative Regulator (PTGFRN) as an internalizable target in cancer cells for antibody-drug conjugate development. PLoS ONE 16:e0246197. 10.1371/journal.pone.0246197 33503070 10.1371/journal.pone.0246197 PMC7840024

[37] Rhodes DR, Kalyana-Sundaram S, Mahavisno V, Varambally R, Yu J, Briggs BB, Barrette TR, Anstet MJ, Kincead-Beal C, Kulkarni P, Varambally S, Ghosh D, Chinnaiyan AM (2007) Oncomine 3.0: genes, pathways, and networks in a collection of 18,000 cancer gene expression profiles. Neoplasia 9:166–180. 10.1593/neo.07112 17356713 10.1593/neo.07112 PMC1813932

[38] Brown CE, Alizadeh D, Starr R, Weng L, Wagner JR, Naranjo A, Ostberg JR, Blanchard MS, Kilpatrick J, Simpson J, Kurien A, Priceman SJ, Wang X, Harshbarger TL, D’Apuzzo M, Ressler JA, Jensen MC, Barish ME, Chen M, Portnow J, Forman SJ, Badie B (2016) Regression of glioblastoma after chimeric antigen receptor T-cell therapy. N Engl J Med 375:2561–2569. 10.1056/NEJMoa1610497 28029927 10.1056/NEJMoa1610497 PMC5390684

[39] Priceman SJ, Tilakawardane D, Jeang B, Aguilar B, Murad JP, Park AK, Chang WC, Ostberg JR, Neman J, Jandial R, Portnow J, Forman SJ, Brown CE (2018) Regional delivery of chimeric antigen receptor-engineered T cells effectively targets HER2(+) breast cancer metastasis to the brain. Clin Cancer Res 24:95–105. 10.1158/1078-0432.Ccr-17-2041 29061641 10.1158/1078-0432.CCR-17-2041 PMC7685198

[40] Wang X, Huynh C, Urak R, Weng L, Walter M, Lim L, Vyas V, Chang WC, Aguilar B, Brito A, Sarkissian A, Bandara NA, Yang L, Wang J, Wu X, Zhang J, Priceman SJ, Qin H, Kwak LW, Budde LE, Thomas SH, Clark MC, Popplewell L, Siddiqi T, Brown CE, Forman SJ (2021) The cerebroventricular environment modifies CAR T cells for potent activity against both central nervous system and systemic lymphoma. Cancer Immunol Res 9:75–88. 10.1158/2326-6066.Cir-20-0236 33093217 10.1158/2326-6066.CIR-20-0236 PMC8008993

[41] Yaghoubi SS, Jensen MC, Satyamurthy N, Budhiraja S, Paik D, Czernin J, Gambhir SS (2009) Noninvasive detection of therapeutic cytolytic T cells with 18F-FHBG PET in a patient with glioma. Nat Clin Pract Oncol 6:53–58. 10.1038/ncponc1278 19015650 10.1038/ncponc1278 PMC3526373

[42] Brown CE, Hibbard JC, Alizadeh D, Blanchard MS, Natri HM, Wang D, Ostberg JR, Aguilar B, Wagner JR, Paul JA, Starr R, Wong RA, Chen W, Shulkin N, Aftabizadeh M, Filippov A, Chaudhry A, Ressler JA, Kilpatrick J, Myers-McNamara P, Chen M, Wang LD, Rockne RC, Georges J, Portnow J, Barish ME, D’Apuzzo M, Banovich NE, Forman SJ, Badie B (2024) Locoregional delivery of IL-13Rα2-targeting CAR-T cells in recurrent high-grade glioma: a phase 1 trial. Nat Med 30:1001–1012. 10.1038/s41591-024-02875-1 38454126 10.1038/s41591-024-02875-1 PMC11031404

[43] Liu Z, Zhou J, Yang X, Liu Y, Zou C, Lv W, Chen C, Cheng KK, Chen T, Chang LJ, Wu D, Mao J (2023) Safety and antitumor activity of GD2-Specific 4SCAR-T cells in patients with glioblastoma. Mol Cancer 22:3. 10.1186/s12943-022-01711-9 36617554 10.1186/s12943-022-01711-9 PMC9827625

