# Supplementary material for: Prostaglandin F2 receptor negative regulator as a potential target for chimeric antigen receptor-T cell therapy for glioblastoma
Source: Cancer Immunol Immunother. 2025 Mar 6;74(4):136. doi: 10.1007/s00262-025-03979-4 (PMC11885767; doi:10.1007/s00262-025-03979-4)
Supplement: Supplementary file 1 — Supplementary file1 (PDF 2012 KB) [file 262_2025_3979_MOESM1_ESM.pdf]

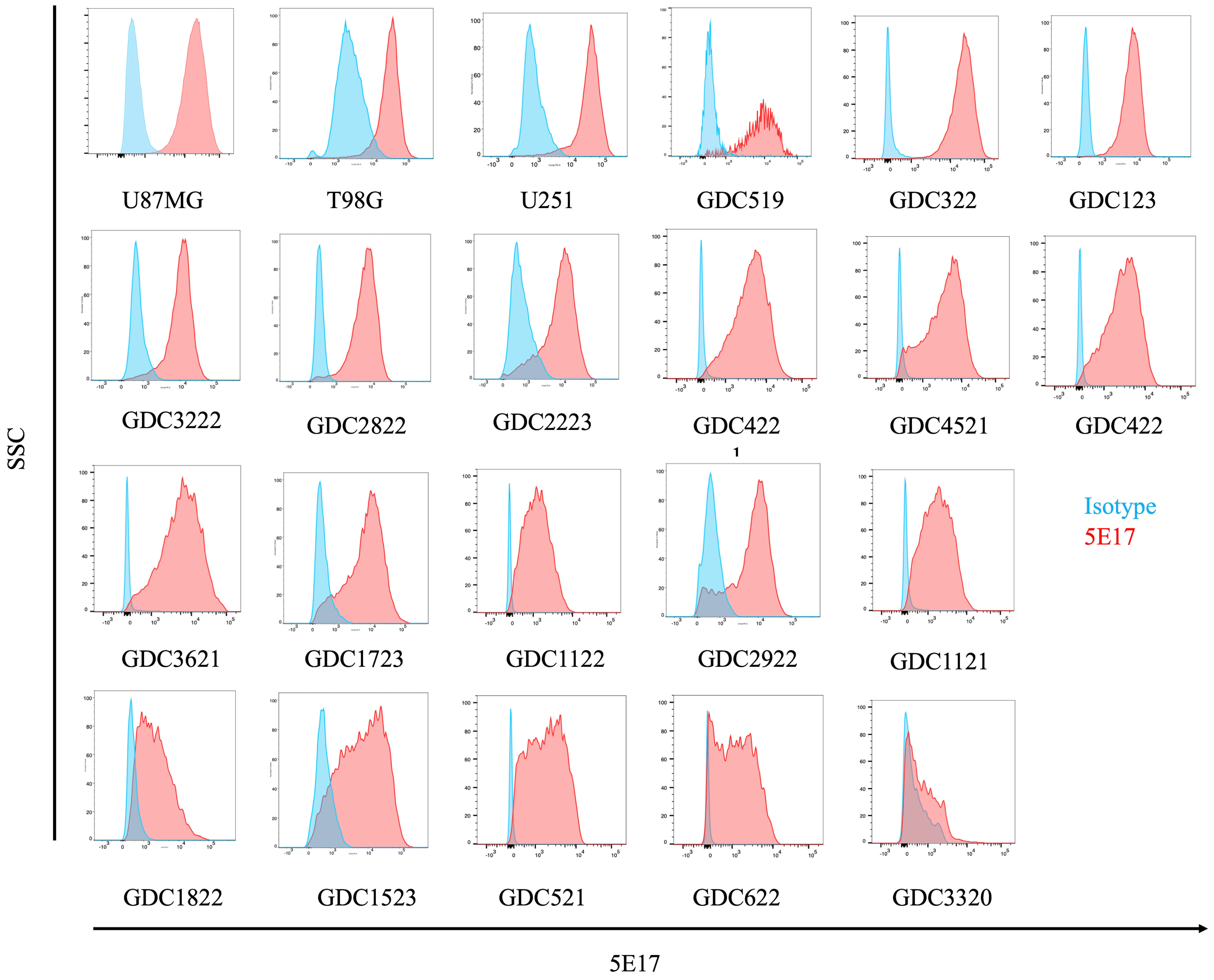

Supplementary Figure1: Flow cytometry analysis of 5E17 reactivity to GBM cells

Flow cytometric analyses of 5E17 reactivity in GBM cell lines (U87MG, T98G and U251) and patient-derived tumor cell lines (others). The result of staining with the isotype instead of anti-5E17 monoclonal antibody were used to draw the gate for 5E17-positive cells. Blue histogram indicates isotype control. SSC, side scatter.
