# Supplementary material for: Prostaglandin F2 receptor negative regulator as a potential target for chimeric antigen receptor-T cell therapy for glioblastoma
Source: Cancer Immunol Immunother. 2025 Mar 6;74(4):136. doi: 10.1007/s00262-025-03979-4 (PMC11885767; doi:10.1007/s00262-025-03979-4)
Supplement: Supplementary file 2 — Supplementary file2 (PDF 2352 KB) [file 262_2025_3979_MOESM2_ESM.pdf]

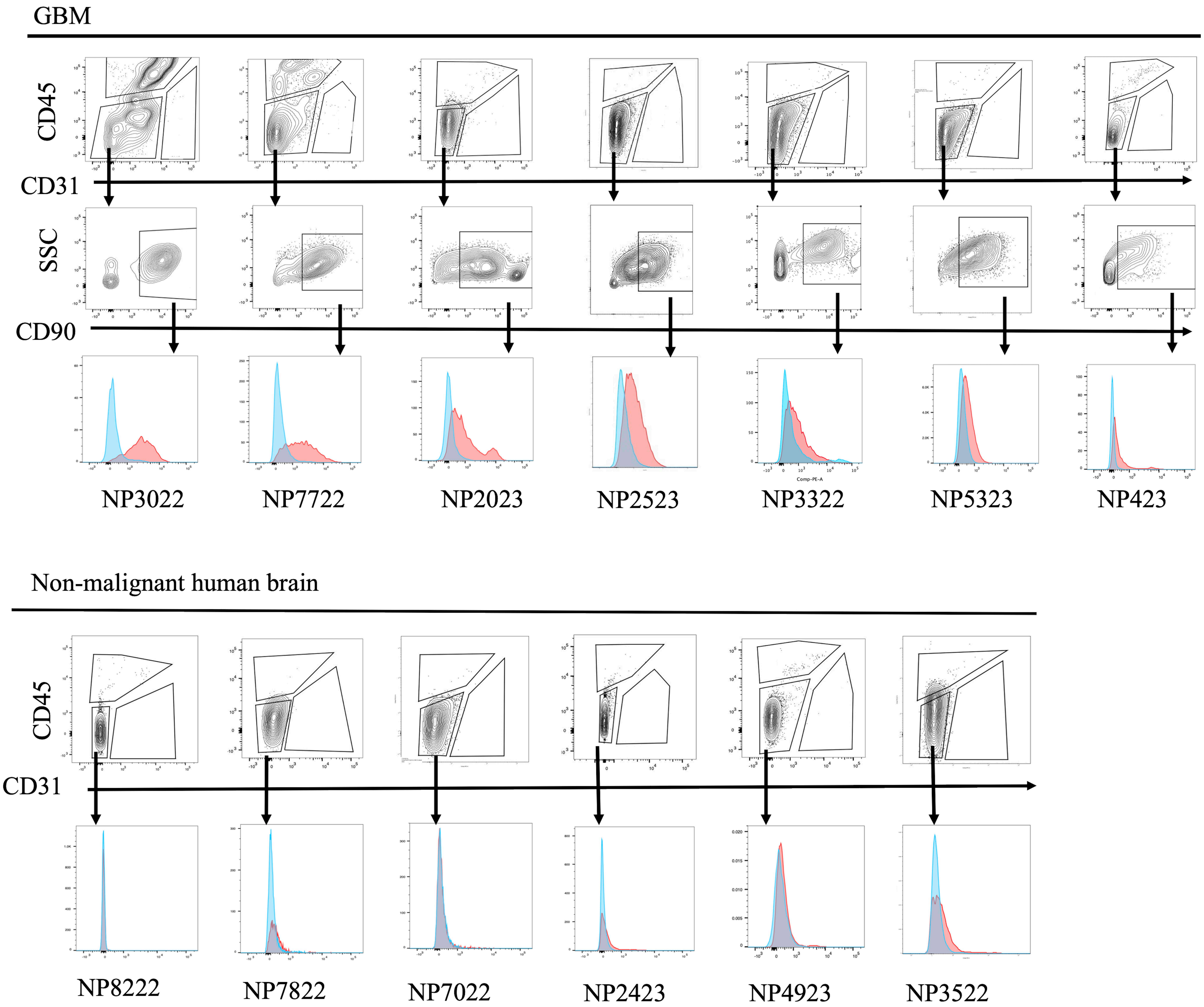

Supplementary Figure 2: Flow cytometry analysis of 5e17 reactivity to surgical tissue samples

Flow cytometry analysis of 5E17 reactivity to CD31-CD45-CD90+ GBM tumor cell from patients with GBM and CD30-CD45- nonmalignant human brain cells from patients with epilepsy. The results of staining with the isotype instead of anti-5E17 monoclonal antibody were used to draw the gate for 5E17-positive cells. Blue histogram indicates isotype control. SSC, side scatter.
