# Supplementary material for: Prostaglandin F2 receptor negative regulator as a potential target for chimeric antigen receptor-T cell therapy for glioblastoma
Source: Cancer Immunol Immunother. 2025 Mar 6;74(4):136. doi: 10.1007/s00262-025-03979-4 (PMC11885767; doi:10.1007/s00262-025-03979-4)
Supplement: Supplementary file 3 — Supplementary file3 (PDF 378 KB) [file 262_2025_3979_MOESM3_ESM.pdf]

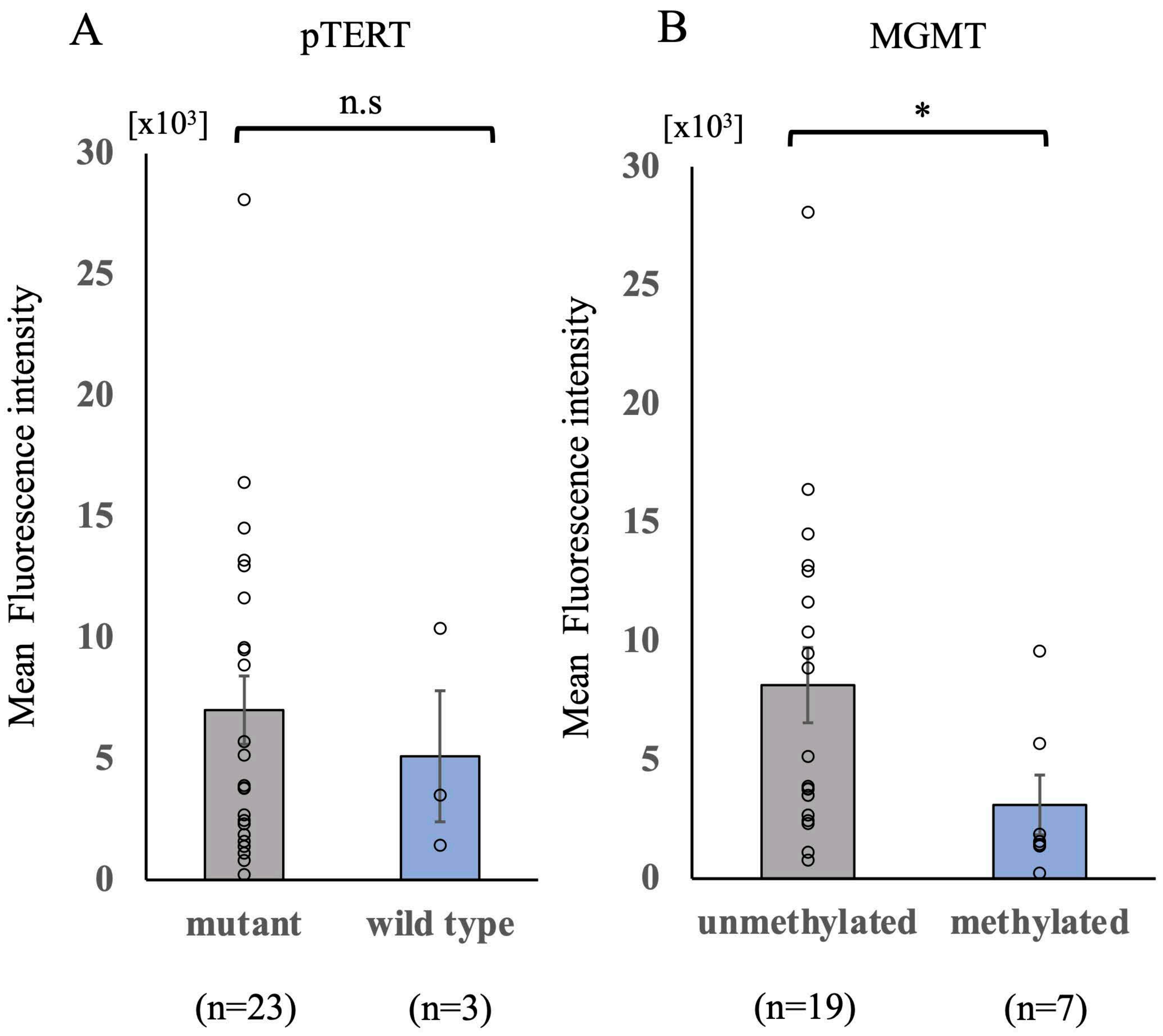

Supplementary Figure 3: 5E17 reactivity in PDTCs and primary GBM samples by gene

Mean fluorescence intensity (means ± standard error of the mean) of flow cytometry findings bound to GBM from patients have either pTERT mutation or not (A) or MGMT methylation or not(B). Each plot shows the mean fluorescence intensity. pTERT, TERT promotor. n.s, not significant. * $p< 0.05$, calculated using the Mann-Whitney U-test
