# Supplementary material for: Prostaglandin F2 receptor negative regulator as a potential target for chimeric antigen receptor-T cell therapy for glioblastoma
Source: Cancer Immunol Immunother. 2025 Mar 6;74(4):136. doi: 10.1007/s00262-025-03979-4 (PMC11885767; doi:10.1007/s00262-025-03979-4)
Supplement: Supplementary file 4 — Supplementary file4 (PDF 1417 KB) [file 262_2025_3979_MOESM4_ESM.pdf]

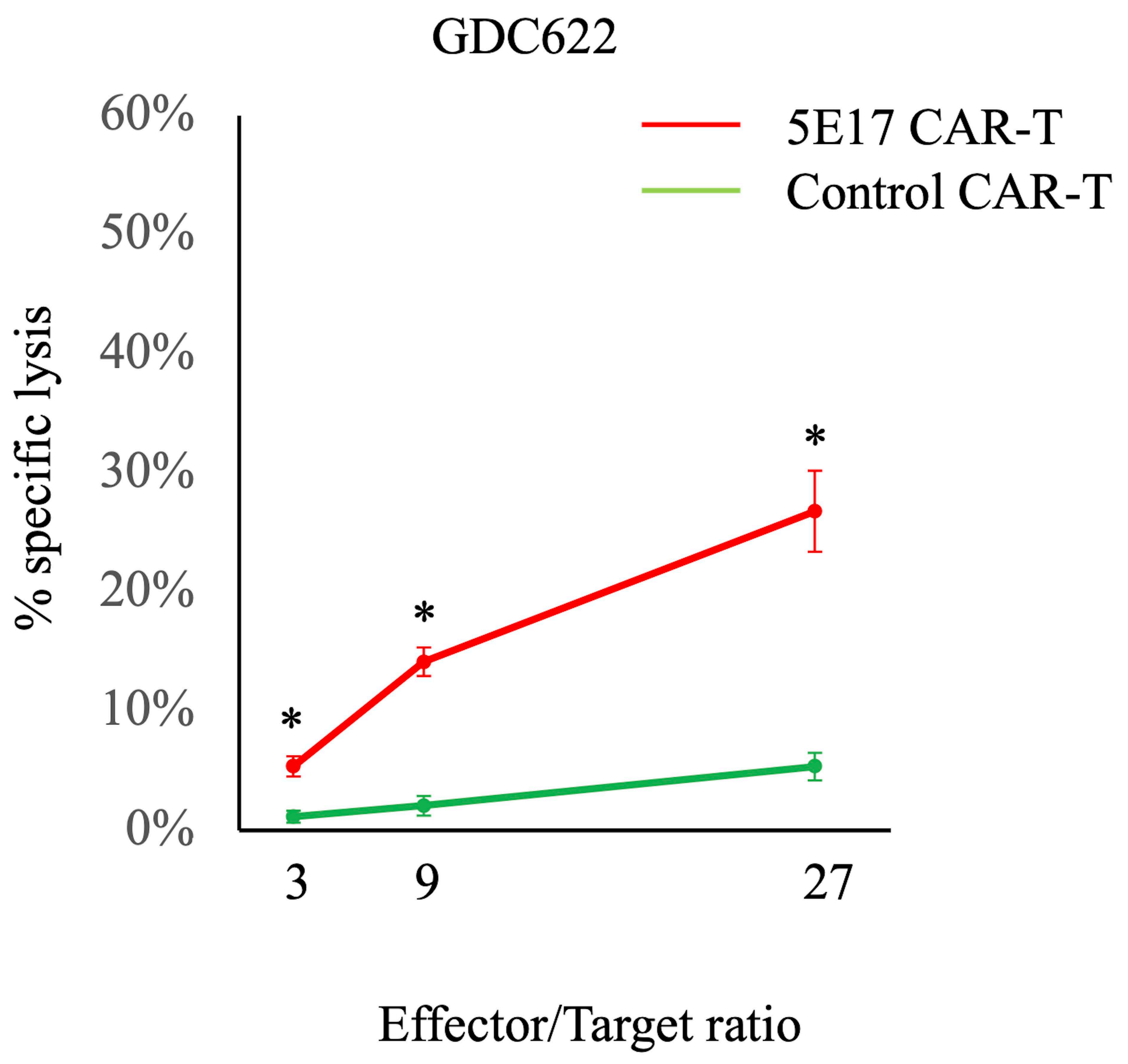

Supplementary Figure 4: Cytotoxicity in cells with low 5E17 reactivity

Assay of ^{51}Cr release to measure specific lysis of GDC622 cells by 5E17 or control CAR-T cells. All experiments were performed in technical-triplicate wells. Data are expressed as means ± standard error of the means. * *p< 0.05*, calculated using the Mann-Whitney U-test
